# Are we getting enough sulfur in our diet?

**DOI:** 10.1186/1743-7075-4-24

**Published:** 2007-11-06

**Authors:** Marcel E Nimni, Bo Han, Fabiola Cordoba

**Affiliations:** 1 Departments of Surgery and Biochemistry and Molecular Biology, Keck School of Medicine, University of Southern California, Los Angeles, CA. 90032, USA; 2 Pediatrics Medical Group, San Juan, 00907, Puerto Rico

## Abstract

Sulfur, after calcium and phosphorus, is the most abundant mineral element found in our body. It is available to us in our diets, derived almost exclusively from proteins, and yet only 2 of the 20 amino acids normally present in proteins contains sulfur. One of these amino acids, methionine, cannot be synthesized by our bodies and therefore has to be supplied by the diet. Cysteine, another sulfur containing amino acid, and a large number of key metabolic intermediates essential for life, are synthesized by us, but the process requires a steady supply of sulfur.

Proteins contain between 3 and 6% of sulfur amino acids. A very small percentage of sulfur comes in the form of inorganic sulfates and other forms of organic sulfur present in foods such as garlic, onion, broccoli, etc.

The minimal requirements (RDA) for all the essential amino acids have always been estimated in terms of their ability to maintain a nitrogen balance. This method asses amino acid requirements for protein synthesis, only one of the pathways that methionine follows after ingestion. To adequately evaluate the RDA for methionine, one should perform, together with a nitrogen balance a sulfur balance, something never done, neither in humans nor animals.

With this in mind we decided to evaluate the dietary intake of sulfur (as sulfur amino acids) in a random population and perform sulfur balance studies in a limited number of human volunteers. Initially this was done to try and gain some information on the possible mode of action of a variety of sulfur containing compounds (chondroitin sulfate, glucosamine sulfate, and others, ) used as dietary supplements to treat diseases of the joints. Out of this study came information that suggested that a significant proportion of the population that included disproportionally the aged, may not be receiving sufficient sulfur and that these dietary supplements, were very likely exhibiting their pharmacological actions by supplying inorganic sulfur.

## Introduction

Because of the much larger implications of sulfur metabolism, and the role that this element plays in the synthesis of a very large number of key metabolic intermediates, such as glutathione, we decided to extend this review to include a broad scope of overlapping metabolic pathways that can be affected by insufficient or marginal intake of sulfur. The hope is that such a review will encourage further research into this very important and most often neglected area of metabolism. This includes the potential to affect the initiation and progression of a large number of anomalies presenting inflammatory and degenerative changes as well as those associated with normal aging and the wasting aspects of a large number of pathologies.

Sulfur containing metabolites, of which glutathione is a key exponent, merge in their functioning with many other compounds that play a major role in mechanisms which are receiving tremendous interests as parts of conventional and complementary medical care. These include the n-3 and n-6 polyunsaturated fatty acids, minerals such as Selenium, Zinc, Copper and Magnesium, vitamins E and C, antioxidants such as the proanthocyanidins and lipoic acid, many of which are involved in the synthesis of prostaglandins and in the antioxidant cascade. More and more evidence is accumulating and focusing on the cooperative role that glutathione and other sulfur metabolites play in the homeostatic control of these fundamental mechanisms.

### Metabolism of sulfur containing amino acids

Methionine and cysteine are both required for protein synthesis by simple-stomached mammals and avian species [1]. For optimal growth, diets must provide these two amino acids, or methionine alone. The physiological requirements for cysteine can be met by dietary cysteine or by an excess of dietary methionine. The molar efficiency of trans-sulfuration, i.e. methionine sulfur converted to cysteine sulfur, is 100%. Cysteine can reduce the requirements of dietary methionine even though no cysteine is converted to methionine in higher organisms by sparing its utilization for essential processes. From the standpoint of the diet, methionine alone is capable of providing all the necessary body sulfur, with the exception of the two sulfur-containing vitamins, thiamin and biotin.

In 1989 a subcommittee of the United States Food and Nutrition Board, National Research Council, issued its last update on recommended dietary allowances (RDA) for protein and amino acids (These recommendations rely on N balance studies performed many years ago [2-4]. The RDA for methionine (combined with cysteine) for adults has been set at 14 mg/Kg of body weight per day. Therefore a person weighing 70 Kg, independent of age or sex, requires the consumption of around 1.1 g (0.9 mMoles) of methionine/cysteine per day. When Rose proposed these amounts he suggested that a "safe intake" should be twice that amount or 2.0 g/day, probably acknowledging that his studies had been done on limited numbers of individuals, usually 3–6 for each amino acid.

These requirements of man for methionine, and the sparing effects of cysteine, determined in young healthy volunteers in 1955 by Rose et al are still accepted today, in spite of indications that they may not represent universal values [5]. Tuttle et al [6] feeding purified amino acid diets containing variable amounts of methionine to older individuals at the VA Hospital in Los Angeles/UCLA established values significantly higher than those previously established by Rose in young college students. They all needed more than 2.1 g/day, with some subjects requiring up to 3.0 g/day to remain in positive nitrogen balance. Although Fukagawa et al [7] were not able to confirm such differences using amino acid oxidation, rather that N-balance as criteria; they agreed that further studies were required. Neither their approaches, which relies on the production of isotope enriched CO2, nor the nitrogen balance studies take into account the unique role of the SAA (sulfur amino acids), to provide S for sulfation. Fuller and Garlick [8] who reviewed the subject in detail concluded that, both for men and women amino acid requirements appear underestimated.

In light of these concerns, particularly as it relates to the unique role of the SAA in providing sulfates for GAG (glycosaminoglycans) synthesis, it seems essential to determine if the needs for sulfur are being met, in particular as it relates to GAG and GSH (glutathione) in cartilage. One could predict that GAG synthesis may not fare well during marginal intakes, and that a preference will be given to the synthesis of proteins and essential metabolic intermediates like CoA, SAM (S-Adenosyl-L-Methionine), GSH, etc. in the brain and other fundamental organs. Unfortunately no studies have been performed to address this very important question.

Studies in humans are not easy to perform, are costly and subject to many variables. In other species, there appears to be more information, particularly in poultry or cattle, where growth stimulation represents a significant economic advantage. It should be noted that poultry diets are always supplemented with methionine/cysteine to enhance growth [9,10].

### Factors that can reduce the availability of methionine/cysteine

Sulfation is a major pathway for detoxificication of pharmacological agents by the liver. As already mentioned, certain drugs that play a key role in the treatment of cartilage anomalies, such as acetaminophen require sulfate for their excretion. Acetaminophen is given in large doses to alleviate pain, and doses of up to 4 gm/day are recommended in the label, but often more is consumed. Thirty five % is excreted conjugated with sulfate and 3% conjugated with cysteine [11]. The rest is excreted conjugated with glucuronic acid, incidentally also one of the major components of GAG.

Methionine or cysteine (0.5%) added to the diet can overcome the severe methionine deficiency induced in rats by the addition of 1% acetaminophen, (an equivalent to the 4 gm/day to a human dose) [12]. It is interesting to note that D- as well as L-methionine could restore growth, implying that depletion of sulfur was the primary defect and not one related to protein synthesis.

**Figure 1:**
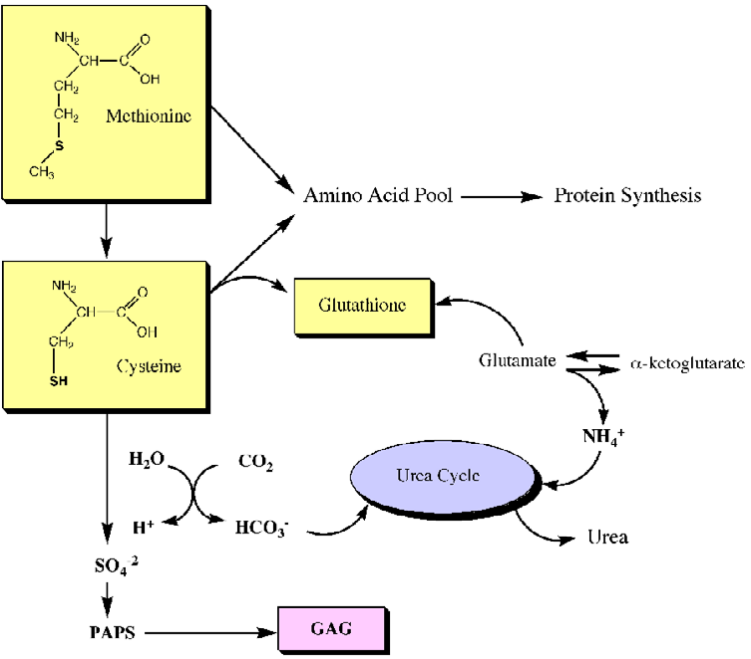
Simplified diagram that depicts the relationships between SAA, GAG synthesis, storage of cysteine as glutathione, protein synthesis and nitrogen metabolism.

Most important is that hepatic concentrations of active sulfate, in the form of PAPS (adenosine 3'-phosphate 5'-phosphosulftate) a key metabolic precursor of GAG was also decreased and could be restored to normal by supplementation with methionine [13]. Urinary sulfate excretion was reduced up to 95% by feeding low-methionine diets to rats, and a 60% decrease in liver methionine was observed, [14]. Depending on the degree of depletion restoration of normal sulfate excretion and levels of liver glutathione could be achieved during supplementation. Inorganic sulfate was not as effective in restoring PAPS levels as methionine (Fig 1).

In rats exposed to a deficient intake of sulfur to study sulfation of acetaminophen for purpose of biodegradation alterations in PAPS homeostasis were observed [15]. Concomitantly these animals eliminated acetaminophen from blood at a slower rate and converted it to a toxic thioether intermediate. Reduced sulfation appears to be caused by decreased availability of inorganic sulfate for PAPS synthesis.

### Glutathione (GSH) a key metabolite and storage form for sulfur

Sulfur amino acids contribute substantially to the maintenance and integrity of the cellular systems by influencing cellular redox state and the capacity to detoxify toxic compounds, free radicals and reactive oxygen species [16]. Cysteine and methionine are not stored in the body. Any dietary excess is readily oxidized to sulfate, excreted in the urine (or reabsorbed depending on dietary levels) or stored in the form of glutathione (GSH). Even in extreme situations, such as when tryptophane deficiency leads to a general catabolic effect, the organism tries to spare the loss of sulfur by continuing to store any available sulfur as GSH in the liver [17]. The availability of cysteine appears to be the rate limiting factor for synthesis of GSH. GSH values are subnormal in a large number of wasting diseases and following certain medications leading frequently to poor survival [18,19]. By supplying SAA many of these changes can be reversed. In the brain, which is usually the most spared organ during nutrient deficiencies, GSH concentration declines in order to maintain adequate levels of cysteine. This loss of GSH impairs antioxidant defenses. The active form of glutathione is the reduced form, GSH, while the inactive form GSSG, has to be converted to GSH. The usual ratio of GSH:GSSG in tissues is around 100:1. Cartilage, less essential for survival, may not fare well under conditions of sulfur deprivation, explaining why dietary supplements containing sulfur (chondroitin sulfate, glucosamine sulfate, MSM (Methylsulfonylmethane), etc.) may be of benefit in the treatment of joint diseases [20]. Neither GSH nor GAG synthesis have been investigated in this context.

Even sulfurated water hydrotherapy, many times accompanied by the ingestion of such waters, and considered an empirical treatment for a variety of diseases, has been shown to involve the GSH related antioxidant cascade [21,22]. The relationship of diet, age and other physiological parameters to blood and tissue concentrations of GSH are well documented [23-26]. Since all the dietary supplements investigated containing sulfate, including MSM [27] are readily metabolized prior or shortly after absorption to sulfate or small molecular weight intermediates, they should be able to spare losses of GSH associated with dietary deficiencies, increased utilization due to disease or altered immune function.

Reactive oxygen species (ROS) are generated during normal cellular activity and may exist in excess in some pathophysiological conditions, such as inflammation or preperfusion injury. These molecules oxidize a variety of cellular constituents, but sulfur-containing amino acid residues are especially susceptible [28]. Therefore exploring fundamental aspects of sulfur metabolism such as the regulation of cell function by methionine oxidation and reduction and the antioxidant effects of sulfur-containing amino acids [29] may help elucidate the mechanism by which the dietary supplements in question work.

### Glutathione: its protective role against oxidative and free radical damage and its potential to enhance the immune function

The manner in which cells and tissues respond to variations in SAA intake is constrained by the characteristics of the key enzymes in the metabolic pathways involved [30]. At low intracellular concentrations of methionine, remethylation of the metabolic product is favored over transsulfuration, and methionine is conserved. With increasing methionine intake, the transsulfuration pathway, which provides a substrate for GSH synthesis, is increased.

Thus, under conditions of low SAA intake, protein synthesis will be maintained, and synthesis of sulfate and GSH will be curtailed. Changes in the availability of GSH are likely to influence in a negative fashion the function of the immune system and of the antioxidant defense mechanisms.

High dietary intakes methionine (5–6 g/day) on the other hand have been shown to raise plasma levels of homocysteine, despite adequate intakes of B vitamins [31-33]. This raises some concern as one does not want to activate the immune system at the cost of enhancing monocyte adherence to endothelial cells.

As discussed earlier GSH is influenced by dietary SAA intake. In an isotopic study in rats, when diets with various SAA contents were fed at adequate levels, 7 molecules of S were incorporated into GSH for every 10 incorporated into protein [34]. At inadequate levels of intake the ratio fell to <3:10. This response to a low intake of SAA is causes antioxidant defenses to become compromised.

A reduction in the levels of GSH, and consequently of antioxidant defenses, may increase the risk of damage to the host via transcription factor activation leading to up-regulation of proinflammatory cytokines, such as nuclear transcription factors and activator proteins, induced in turn by agents such as hydrogen peroxide, mitogens, bacteria, viruses, and UV and ionizing radiation.

Oxidant damage to cells will give rise to a cascade of proinflammatory effects by the production of lipid peroxides. Even though some of these effects are biphasic in nature as it relates to levels of SAA, it is generally accepted that GSH and the associated antioxidant activity exerts an immune enhancing effect by activating transcription factors that are strongly associated with cell proliferation as well as a parallel an anti-inflammatory effect as described earlier.

Further knowledge of these metabolic processes, at other levels beyond the availability of substrate, will be necessary for us to be able to modulate with more accuracy these events to the benefit of the whole organism.

### Regulation of prostaglandin biosynthesis by glutathione

Prostaglandins (PGs) are very well known to play an important role in a variety of normal body functions as well in key metabolic steps associated with many of the events associated with inflammation.

PGs are synthesized from free arachidonic acid via two isoforms of cyclooxygenase (COX, also referred to as PGH2 synthetase). Although the availability of arachidonic acid has been actively investigated as a major metabolic factor controlling PG production, it is clear that other cellular cofactors may also regulate PG biosynthesis.

PGH synthetase has two activities, the cyclooxygenase activity which introduces the 5-membered ring into the PUFA (polyunsaturated fatty acid), and another which introduces an endoperoxide and a hydroperoxide into the PUFA. The peroxidase activity reduces the hydroperoxide into a hydroxyl group using GSH as a source of reducing equivalents.

**Figure 2:**
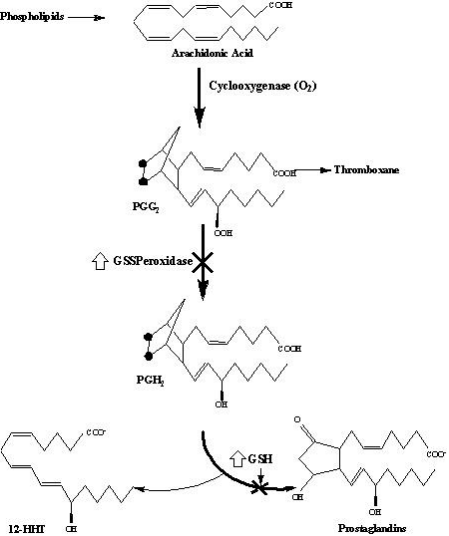
Enzymatic conversion of arachidonic acid (AA) to PGs and the sites of inhibition by GSH and GSH peroxidase (GSSPx). (Adapted from Marglit et al. [36]).

The observation that both the constitutive and the mitogen inducible isoforms of prostaglandin H2 synthetase are markedly affected by GSH and GSH peroxidase [35], has catalyzed significant interest in connection with this process. The sites of action of GSH and GSH peroxidase on the metabolic pathway in question is shown in Fig 2.

Studies by Margalit et al [36] has provided clear evidence in mice that elevated levels of GSH inhibit PG production, and most likely exhibit its anti-inflammatory effects in a urate crystal induced model via this mechanism. This attenuation of PG synthesis in vivo sheds light on another potential benefit associated with increased SAA intake and adequate levels of tissue GSH. From the practical point of view, it raises the possibility that a satisfactory intake of SAA combined with PUFA may prove of significant benefit to individuals suffering from a variety of joint anomalies associated with inflammation.

The question of how in the presence of adequate PUFA precursors the constitutive and inducible forms of prostaglandin H synthetase can be induced to generate the appropriate forms of prostaglandin required to maintain tissue homeostasis will rely on further understanding of the co-factors and feedback mechanisms involved.

Until these fundamental questions are answered, the best we can do is to continue to reduce the ratio of omega 6/omega 3 in our dietary supply of PUFA (which currently is around 10.0 compared to 12.0 just a few years ago) by increasing consumption of fish and certain plant oils, to what is considered an ideal ratio of 2.3/1.0 [37].

### Metabolic studies exploring the relationship of dietary sulfur intake to urinary excretion of inorganic sulfate

The lack of available data on the relationship of dietary supplements, such as chondroitin sulfate and its analogues, to dietary intake of sulfur and to any possible relationship encouraged us to contemplate a series of human metabolic/balance studies that would relate intake of proteins, dietary supplements containing sulfur and SAA to sulfate excretion.

**Figure 3:**
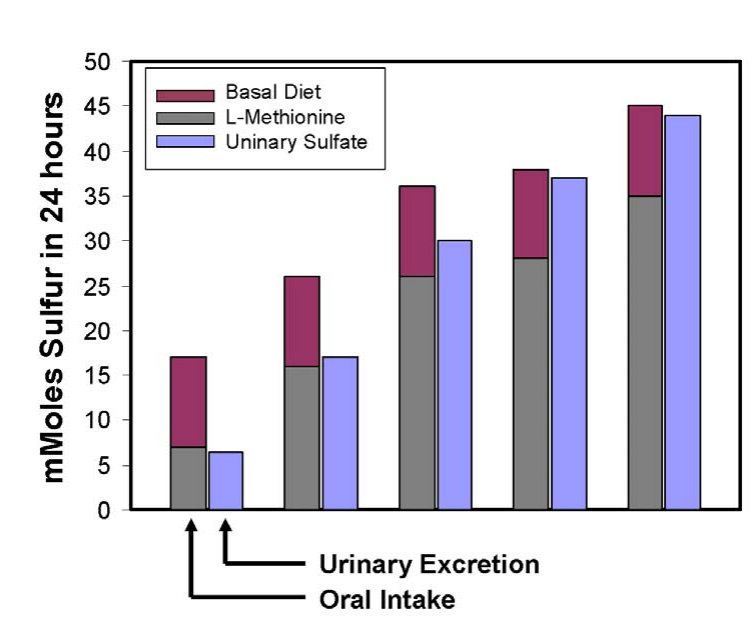
Intake of SAA as part of the basic diet (dark bar) are superimposed by a 10 mmole supplement of methionine administered as a single dose on the morning of the experiment. The total height of the bar therefore represents intake of S in mmoles. In an adjacent bar is the amount of free sulfate excreted in the urine over a 24 hour period.

Our preliminary studies were presented at the American College of Clinical Nutrition, American College of Rheumatology (2001) and subsequently published [20]. These studies, even though limited in scope, provide further evidence for the ready conversion of sulfur in dietary supplements into inorganic sulfate. The retention of sulfur from SAA or from the dietary supplements administered during the ingestion of low or marginal levels of protein as compared to the enhanced excretion during higher levels of intake provided valuable clues (Fig. 3). It would appear from our findings that the minimum adequate intake values determined in a VA setting in older people by Tuttle et al [6] may closer to being accurate than those currently accepted as the RDA.

Our studies were performed in normal volunteers (ages 35–70). Sulfate, free and esterified were measured by a modification of the nephelometric method of Berglund and Sorbo [38] and urine creatinine using a kit (555-A) from Sigma. Intake of SAA was evaluated with the aid of a Nutritional Analysis Software (ESHA Research, version 7.6). L-methionine (Solgar) was purchased as dietary supplements. A sustained release formulation of L-methionine was specially prepared by Xcel Medical Pharmacy (Woodland Hills, CA.).

### Relationship between dietary intake of protein with or without Methionine or S-containing compounds and urinary excretion of free sulfate

Subjects were adapted to a particular level of dietary protein by starting them on their diets 24 hours in advance of the actual test. Protein levels were incremented by the addition of low-fat tuna, which is mostly protein, to the basal diet. Figure 3 summarizes sulfur balance studies that include L-methionine supplements.

Our findings (Fig 3) clearly demonstrate that S retention occurs during the consumption of low levels of protein. When less than 10 mmoles of sulfur derived from dietary proteins are consumed, supplementing the diet with 10 mmoles of L-methionine was accompanied by retention of this amino acid. At higher levels of dietary protein intake, when the requirements of sulfur are presumably met, essentially all the methionine added to the diet is excreted in the urine.

The significant retention of methionine at low levels of protein intake gave the first clues that our dietary supply of sulfur could be borderline or even unsatisfactory for many individuals.

### Intake of SAA in a normal population: relationship to the RDA and to the potential loss of sulfate associated with drug metabolism

A generalized assessment of diet intake and quality is very difficult to make because of obvious reasons. The heterogeneity of populations (cultural, socio-economic, ethnic, geography, occupation, fast-food consumption, advertising, etc) all influence food intake. Nevertheless, it seemed important for the purpose of this study to try and generate a profile that would cover various segments of the population, and relate the values obtained to the accepted RDA for SAA and to the alternate higher requirements suggested by others. To gain further insight we grouped the various individuals evaluated into subgroups (Table 1).

**Table 1:** Average sulfur amino acid intake associated with the consumption of a variety of typical diets.

Group		SAA (g/day)
I	High-protein	6.8
II	High-protein low-calorie	5.0
III	Oriental-American	4.8
IV	Average balanced	4.3
V	Fast-food	4.1
VI	Dieter	3.5
VII	Lacto-ovo-vegetarian	3.0
VIII	"health conscious diet	2.6
IX	Vegan	2.3
X	elderly people (75 yr old)	1.8

**Figure 4:**
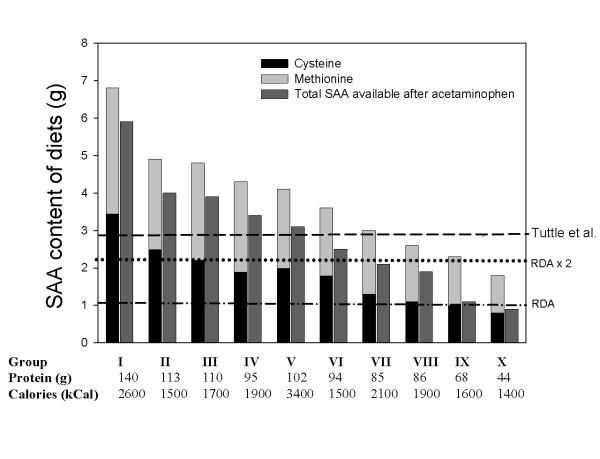
Dietary intake of SAA (methionine plus cysteine) measured in various subgroups of a population. These were compared to suggested requirements: the RDA (1989), 2× the RDA (Rose's safety margin) [4] and Tuttle et al [6] determined in older individuals. A solid bar is included at the right of each group, which represents the SAA intake reduced by 0.9 g/day, to account for the estimated loss of sulfur associated with the consumption of a standard dose of acetaminophen, excreted as a sulfated conjugate.

Even though diets vary periodically we noticed that individuals tend to adopt certain repetitive patterns that in a way facilitated the evaluation. Intake of SAA measured in 32 individuals ranged between 1.8 and 6.0 g/day (14 and 45 mmoles/day). For purposes of calculations the cysteine and methionine were combined as SAA. In general the ratio of cysteine/methionine is close to one for poultry and red meat protein, and to 0.7 for fish. Dairy products tend to have slightly higher levels of methionine and starch rich foods slightly more cysteine. Eggs contain significantly more cysteine. To estimate molar concentrations a 1:1 ratio was employed. Some of the lower SAA values recorded in our survey included individuals who tended to be more health conscious and consume no red meet and little animal protein, as well as those consuming "fad diets". Many older people could turn out to be outright deficient (group X) independent of the criteria used (Fig 4). Obviously these dietary estimates have to be considered very preliminary, but they are meant, at this time, to attempt to shed some light on an area seldom explored.

The above figure compares the SAA intake in g/day to the accepted RDA (1989), twice the RDA values accepted to provide greater safety to a large population, and to values arrived to for older individuals by thee VA study of Tuttle et al. [6].

Also a column is included, in which the available SAA are reduced by 0.9 g/day, equivalent to a SAA loss associated with the intake of the standard higher recommended dose of acetaminophen. As already noted, this drug, as well as several others, is excreted in great part conjugated with sulfate. Depending on which assumption for minimum requirements are used, only those groups who emerge above the cut-off lines would be receiving an adequate amount of SAA. Using Tuttle et al estimates [6] (which agree well with our current estimates) combined with the estimated loss of sulfate due to acetaminophen conjugation, a large segment of the population, which include those most vulnerable to OA, would appear to be sulfur deficient or receiving marginal intakes.

At this time we cannot draw any solid conclusions from these estimates. We know that renal re-absorption of sulfates increases during periods of deficiency [39] but not how long such a sparing effect can hold. The values obtained by Tuttle et al are derived from a limited selected VA population. So are ours as well as those of Rose et al. Until these studies are expanded to include simultaneous S and N-Balance determinations and biosynthetic studies in animals, and well controlled S-balance studies in humans are performed we will not be able to clearly answer this important question.

**Figure 5:**
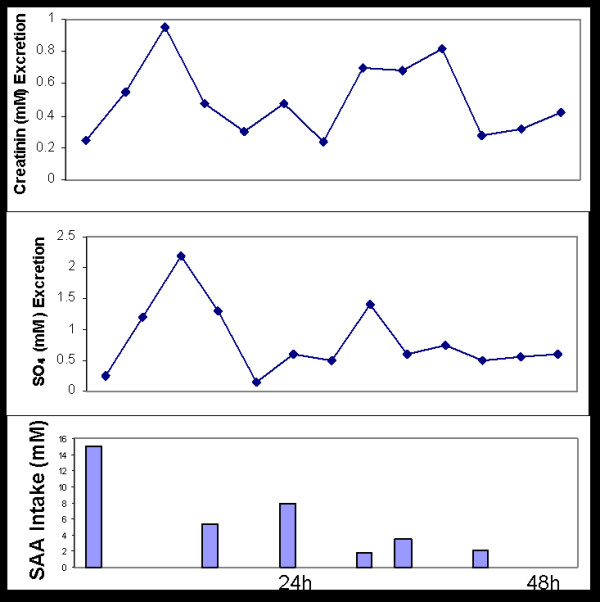
Urinary excretion of sulfates and creatinine during consumption of a standard diet, over a period of 48 hours.

It should be pointed out that we could not find in the recorded literature any studies that effectively measure sulfur balance in human or other animals. All metabolic studies in this connection, even those that focus on the requirements for sulfur amino acids, study nitrogen balance but not sulfur balance. Essentially this means that the role of sulfur amino acids has only been evaluated in herms of protein synthesis, but never in terms of their ability to contribute sulfur to so many important metabolites. As part of our preliminary investigations we evaluated the dietary intake of SAA, the urinary excretion of inorganic sulfate and of creatinine by a 35 year old male subject consuming a random balanced diet over a 3 day period. Results are summarized in Fig 5.

The above figure emphasizes another aspect of the proposed studies, the relationship between S and N excretion. We did not include the ethereal sulfate (less than 5% of total) since no medication was being consumed. Excretion of creatinine over a 24 hour periods has for a long time been related to muscle mass and used for metabolic calculations. They are not useful for N- balance studies since they do not follow protein intake [40]. On the other hand it is clear that sulfate intake and excretion correlate quite well. Free amino acids in general cannot be stored and the SH moiety of cysteine, in particular, is readily oxidized. Cysteine can be cytotoxic since the reactive thiol-amine structure can combine with aldehydes such as pyridoxal, and can also chelate essential divalent cations. SAA are used to replenish the stores of GSH, which can be considered a storage form for sulfur, and only when this goal is met is the excess oxidized to sulfate.

**Figure 6:**
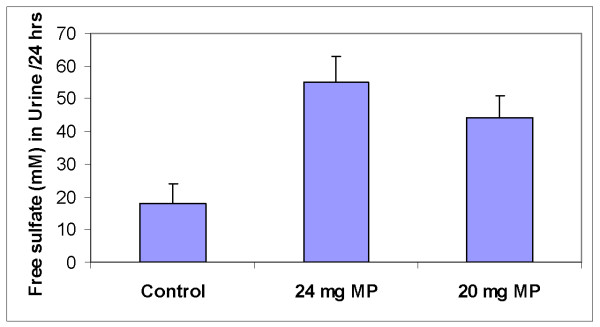
Urinary excretion of free sulfate following a single oral dose of methyl prednisolone (24 mg) followed by a second dose of (20 mg) the following day, while ingesting a diet that supplied 19 mmoles of SAA/day.

The excretion of sulfate associated with the administration of methyl prednisolone is included to illustrate how a catabolic event can affect sulfur loss (Fig 6). Since steroids are frequently used by patients with joint diseases, the large excretion of sulfate may, among other things, interfere with PG synthesis and other important metabolites such as GSH. This aspect of the catabolic effect of steroids does not seem to have been explored.

**Figure 7:**
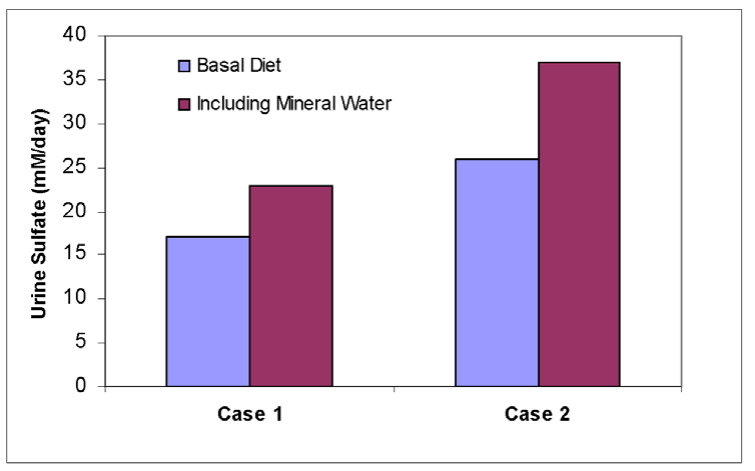
Twenty four hour urinary excretion of sulfate by individuals consuming different amounts of protein combined with 10 mmoles of sulfate from a mineral water source, evenly distributed through out the daytime hours, and compared to control (case 1, basal diet: 17 mM dietary SAA, case 2, basal diet: 26 mM SAA).

Consumption of sparkling mineral water containing 0.5 g of sulfate/liter (in this case San Pellegrino, one of the very few mineral waters that contains sulfate ions) throughout the day (2 liters containing approximately 10 mmoles) was accompanied by quantitative excretion of sulfate when dietary protein levels supplied 25 mmoles of SAA or more per day (Fig 7).

These findings support the observation, sometimes disputed, that inorganic sulfates are readily absorbed and excreted in urine, in spite of the osmotic effects that they can generate and which lead to their use as laxatives [11,41]. In our case, steady administration of a dilute solution may have facilitated absorption from the GI tract and enhanced urinary excretion. Absorption in the small intestine is estimated to reach 5 mmoles/day, and the remainder is absorbed in the colon [41].

Levels of sulfates in drinking water vary considerably with their source and location. A study in Ohio, evaluating sulfate concentrations in well waters consumed by farm animals, revealed values that ranged between 6 and 1,600 g/liter. The water in our studies, San Pellegrino, from an Italian source is stated in the label to contain .535 g sulfate ions per liter. We found experimentally that the batches used did not vary more than 5% from the stated value. Inorganic sulfates are only very minor components of our diet. Some processed or enriched foods contain minute amounts of sulfites as preservatives and certain additives included in flour, for instance, (ferric sulfate) can contain sulfate. Garlic, onions, and brussel sprouts contain significant amounts of sulfur. Feedstuffs fed to animals has been investigated for their sulfur content in much more detail than foods for humans, and ranges from 0.2% in beet pulp to 1.2% in canola meal (on a dry weight basis) have been observed. As noted earlier the levels of sulfur in the diet can greatly affect the growth and health of livestock.

### Protein and amino acid requirements of the elderly with special focus on sulfur containing amino acids

The estimated protein requirements, for all ages, as discussed earlier, has been based on nitrogen equilibrium studies, and aided occasionally by functional indicators such as immune function or muscle strength. Data from various sources, suggests that the protein requirements for nitrogen equilibrium in the elderly is greater than the 0.8 gm/kg body weight/day. Values of around 1.0 gm/Kg have been suggested [42]. However because of methodological difficulties the data does not allow for a very confident prediction.

As pointed out by Young [43] our knowledge of the dietary requirements for older individuals are often limited and contradictory, although significant efforts continue to be made to overcome this problem. There is a general consensus that the protein requirements of this population are underestimated and it was suggested that a total of 15% of the energy requirements of this population be supplied by proteins. This would translate into an average of around 75–85 g of protein/day. This amount of protein, would supply approximately 3.5 to 4.0 g of SAA per day, which should more than meet all estimates for the requirements of these amino acids. Unfortunately these levels of protein intake are infrequently met by the older segment of the population. The well established sarcopenia associated with older age seems to be associated in part to a decline in protein and energy intake, caused by changes in taste sensation, alterations in dentition, social isolation, depression and economic factors. In addition to the less than optimal food intake, older individuals appear to substitute protein in preference to fat and carbohydrate rich meals, which may again reflect changes in taste [44]. The WHO recommendations for SAA intake of 13 mg/kg of body weight are in the same range as those suggested by the RDA. There is a consensus that in diseases and following trauma these values may be 2 or 3 times higher [45].

There is a growing body of data pointing out the potential importance of oxidative stress and resulting changes in redox state in numerous diseases, including sepsis, chronic inflammation, cancer AIDS/HIV, and of course aging. These observations warrant continued attention for the potential supplementation role of SAA supplementation, in the form of additional protein or as has been found useful, N-acetylcysteine in some particular circumstances. Because of the toxicity of cysteine, and possibly even of methionine supplements given in excess, and the intrinsic problems associated with an induced amino acid imbalance, proteins rich in SAA have been considered as supplements. Immunocal^®^, a purified milk fraction enriched in whey protein is currently being used because of its potential to augment antioxidant defenses and improve immune function [46]. Twenty grams/day of Immunocal significantly enhanced muscular performance and lymphocyte GSH in a group of 20 young adults. Although whey proteins contain significantly more cysteine than casein (2.5% vs. 0.35) the total amount of total SAA is less significantly different (5.2% vs. 3.2%).

Cost and availability become another key factor in reducing dietary protein intake in the aged as do perceived intolerance to certain food groups, difficulty tearing and chewing fibrous foods, as well as the fear of consuming too much fat or cholesterol. Unfortunately eggs, which have a amino acid profile considered as a standard against which other proteins are compared, and which are amongst the proteins with a higher SAA content and are amongst the least expensive in terms of cost, are often not included as major ingredients in the diets of older people.

The importance of dietary protein cannot be underestimated in this population since inadequate protein intake contributes, among other things, to a decrease in lung reserve capacity, increased skin fragility, osteoporosis, decreased immune function and muscle mass (sarcopenia), poor healing and longer recuperation from illness [2,47].

## Discussion

Glutathione (GSH)is the most abundant low molecular weight thiol and form of storage of SH-. Animal and human studies have demonstrated that adequate protein nutrition is crucial for the maintenance of GSH homeostasis [48]. Elevated levels of GSH inhibit prostaglandin production by a direct interaction with COX enzymes, of potential significance in the progression of inflammatory or degenerative states [36]. It is of particular interest, as discussed earlier that prostagandins synthesized from PUFA and most of the non-steroidal anti-inflammatory drugs share this same locus of involvement. It is also relevant that some recent studies have found that on occasions the pain reduction in OA associated with the administration of chondroitin sulfate, a source of sulfur, was found to be equivalent to that provided by NSAID. The reasons for such unpredictable results, we suspect could be associated with differences in levels of protein in the diet, the better responders consuming higher amounts of SAA. This hypothesis will have to be evaluated in future clinical studies.

As discussed neither cysteine nor methionine are stored in the body. Any dietary excess is readily oxidized to sulfate, excreted in the urine (or reabsorbed depending on dietary levels) or stored in the form of glutathione (GSH). Even in extreme situations, such as when tryptophane deficiency leads to a general catabolic effect, the organism tries to spare the loss of sulfur by continuing to store any available sulfur as GSH in the liver. GSH values are subnormal in a large number of wasting diseases and following certain medications, and by supplying SAA many of these changes can be reversed [49]. Whether dietary supplements containing sulfur display similar effects has not been evaluated systematically. Documented improvements in OA and joint pains associated with sulfurated water hydrotherapy, many times accompanied by the simultaneous ingestion of such waters has also been related to the GSH involvement in the antioxidant cascade.

In spite of the apparent complexity associated with evaluating the dietary intake of a population as a whole a pattern seems to emerge, even when evaluating small groups of individuals. In milk and dairy products the methionine/cysteine ratio is around 3/1. It is roughly the same in fishes such as canned tuna, which we used as a source of protein supplement in our studies, and in meats. In eggs, soy beans and other plant products it is around 4/3. The amount of protein in the various foods varies considerably, and the amount of SAA fluctuates. Chicken, fish and beef proteins contain an average of around 5% of SAA. Dairy products, milk, cheese, etc, contain lower levels, around 4%, primarily due to the lower content of SAA in casein. The whey protein fraction, accounts for about 20% of the milk proteins (rich in lactoglobulins) contains more SAA, and is used therapeutically or as a dietary supplement. Plant proteins, in addition to be present in lower amounts, are relatively low in SAA, averaging below 4%. The highest content of SAA is found in egg products, the egg white containing around 8% of SAA.

Consequently the ratios observed in a dietary survey will reflect the amounts of meats, eggs and plant products consumed. The amounts of protein, as a % of the calories consumed, is a major variable in the population. The more weight conscious individuals, and often the ones in more affluent societies, tend to consume less carbohydrate and fats and more proteins. This is counterbalanced some times by the tendency of many to consume less animal products and therefore to include more carbohydrates. In addition the desire to lose weight may reduce both calories and protein intake. Older people, at a time when OA becomes more prevalent, decrease their food intake often at the expense of proteins, frequently due to economic concerns.

Most individuals fall in between the groups established arbitrarily for the purpose of this study, but once a dietary pattern is established deviations are much less than expected. In our experimental studies, the levels of SAA were predetermined and individuals placed on pre-assigned diets containing known amounts of protein. This is critical, since even though the amounts of SAA intake closely reflects the rate of sulfate excretion, below a certain level of intake tubular reabsorption of sulfates prevents further loss. In rats, sulfate renal clearance was significantly decreased in animals that received a low methionine diet, a reflection of a sparing mechanism to retain sulfate [39]. A major unanswered question is how the overall caloric intake affects the requirements of sulfur used for other than protein synthetic purposes, and how long a sparing effect can continue during the prolonged intake of a low protein diet.

Any excess of SAA is oxidized to inorganic sulfate and excrete in the urine as neither organic nor inorganic excesses of sulfur can be stored. The normal concentration of sulfate in serum is around 3.5 mg/100 ml, roughly 5–10% of that as ether sulfate and the rest as sulfate ions. Sulfur is excreted in the urine as it exists in blood.

A deficiency of sulfur amino acids has been shown to compromise glutathione synthesis to a greater extent than protein synthesis in the presence and absence of inflammatory stimulus [34]. During an immune/inflammatory response a combination of enhanced utilization of cysteine for GSH synthesis and cell replication may be what leadsto a depletion of cellular SAM.

In man serum fasting levels of inorganic sulfate were shown to increase with age and exhibit a circadian rhythm, probably associated with food intake. Genetic defects in sulfate transport have been associated with congenital osteochondrodystrophies that may be lethal and provide insights into sulfate transport and hormonal and nutritional regulation [50]. Whereas low levels of dietary protein led to hip joint displasia in mice and rats normal levels inhibited the development of OA.

Even though under normal circumstances dietary inorganic sulfate contributes very little to our sulfate pool, the exogenous administration of small amounts of sulfate in selected forms of delivery may be useful, since contrary to what is still a common belief sulfate can be absorbed form the GI tract [41,51]. Along these lines the possible beneficial effects of inorganic sulfates in drinking water should be evaluated. Certain sulfur containing thermal water baths have been found to be of benefit, probably via transdermal penetration or because of actual drinking of such waters at health spas [21,52-55].

On the other hand it is important to recollect that sulfation is a major pathway for detoxification of pharmacological agents by the liver. Drugs such as acetaminophen, so frequently used in the treatment of pain associated with joint diseases, require large amounts of sulfate for their excretion. Doses of up to 4 g/day are not infrequent. Thirty five % is excreted conjugated with sulfate, 3% conjugated with cysteine [12] and the rest conjugated with glucuronic acid, incidentally a major component of glycosamino glycans (GAG) which are so critical for the integrity of cartilage and other connective tissues.

Methionine or cysteine (0.5%) added to the diet can overcome the severe methionine deficiency induced in rats by the addition of 1% acetaminophen, an equivalent to the 4 g/day of the human dose. D- as well as L-methionine were found to be equally effective, suggesting that depletion of sulfur was at the root of the primary defect and that it was unrelated to protein synthesis. It is well known that N-acetyl-p-benzoquinoneimine, a toxic metabolite of acetaminophen is detoxified by hepatic GSH. Rapid administration of acetyl-cysteine to restore GSH levels remains the treatment of choice following acetaminophen poisoning. Hepatic concentrations of active sulfate, in the form of PAPS (adenosine-3'-phosphate 5'-phosphosulfate) were also decreased and could be restored to normal by supplementation with methionine [13].

The effectiveness of D-methionine in this connection brings back to mind the early studies of Rose who used DL-methionine in his early balance studies which led to the RDA recommendations, again suggesting a significant role for the SAA, beyond that of protein synthesis. That cysteine, sulfite and other sources of sulfates can serve as precursors for GAG synthesis has been well established [56-58]. Also restricting the availability of dietary sulfur in rats (cysteine, sulfate) decreased the biotransformation of acetaminophen, as a consequence of the absence of inorganic sulfate for PAPS synthesis [13,15]. Consequently, addition of a sulfur containing compound to medications such as acetaminophen or catabolic agents such as the corticosteroids, may be a potential way to compensate for sulfur loss.

A major question that arises in connection with dietary supplements that provide organic forms of sulfur, is whether the diet could account for differences in response amongst individuals. It is possible that the individuals that benefit mostly from these supplements are those that consume inadequate amounts of protein or other sources of dietary sulfate. A recent publication by Drogue [59,60], who has extensively investigated the relationship of oxidative stress and aging, has concluded that this event may be in great part be associated with a deficit of cysteine and to a suboptimal intake of SAA.

Finally it may be relevant to conclude this review with a statement taken from Sir Stanley Davidson and Passmore's classic textbook of Human Nutrition and Dietetics [61] who suggested that" it is not unlikely that some of the effects of protein deficiency are in fact due to failure of sulfur containing intermediates or even to sulfur containing polysaccharides. It is even possible that the ancient nostrum of 'brimstone and treacle' (*sulfur and molasses*) had nutritional value unsuspected by modern knowledge".
